# Molecular Epidemiology of *Photobacterium damselae* subsp. *damselae* Outbreaks in Marine Rainbow Trout Farms Reveals Extensive Horizontal Gene Transfer and High Genetic Diversity

**DOI:** 10.3389/fmicb.2018.02155

**Published:** 2018-09-19

**Authors:** Mateus S. Terceti, Ana Vences, Xosé M. Matanza, Inger Dalsgaard, Karl Pedersen, Carlos R. Osorio

**Affiliations:** ^1^Departamento de Microbioloxía e Parasitoloxía, Instituto de Acuicultura, Universidade de Santiago de Compostela, Santiago de Compostela, Spain; ^2^National Institute of Aquatic Resources, Technical University of Denmark, Kongens Lyngby, Denmark; ^3^National Food Institute, Technical University of Denmark, Kongens Lyngby, Denmark

**Keywords:** *Photobacterium damselae*, vibriosis, damselysin, phobalysin, hemolysin, rainbow trout

## Abstract

The marine bacterium *Photobacterium damselae* subsp. *damselae* is a pathogen for a variety of marine animals, as well as for humans, and is nowadays considered an emerging pathogen for fish of importance in marine aquaculture. Recent studies have suggested that outbreaks in fish farms are caused by multiclonal populations of this subspecies that exist in the environment. Here, we report the study of a collection of 31 strains isolated during the course of disease outbreaks in marine rainbow trout farms in Denmark in 1994, 1995, and 2006, respectively. A phylogenetic analysis based on the *toxR* gene sequence, and the screening of virulence-related genes uncovered a high genetic heterogeneity, even among strains isolated from the same fish farm at the same time. Moreover, comparative analysis of the whole genome sequences of four selected strains revealed a large number of differentially occurring genes, which included virulence genes, pPHDD1 plasmid, polysaccharide synthesis gene clusters, CRISPR-Cas systems and putative new mobile genetic elements. This study provides sound evidence that *P. damselae* subsp. *damselae* outbreaks in Danish rainbow trout farms were caused by multiclonal populations and that horizontal gene transfer constitutes a strong driving force in the generation of intraspecific diversity in this pathogen.

## Introduction

The marine bacterium *Photobacterium damselae* subsp. *damselae* has been associated with disease in a number of marine animals, and also in humans. It has been reported as a primary pathogen causing diseases in turbot (*Scophthalmus maximus*) (Fouz et al., 1992), sea bream (*Sparus aurata*) (Vera et al., 1991) and other sparid fish species (Company et al., 1999; Labella et al., 2006), sea bass (*Dicentrarchus labrax*) (Abdel-Aziz et al., 2013; Uzun and Ogut, 2015), rainbow trout (*Oncorhynchus mykiss*) (Pedersen et al., 1997, 2009), shrimp (*Exopalaemon carinicauda*) (Liu et al., 2016), and etc. The geographical distribution of this bacterium is increasing and nowadays it constitutes an emerging pathogen in aquaculture (Abdel-Aziz et al., 2013; Khouadja et al., 2014; Terceti et al., 2016; Sharma et al., 2017; Eissa et al., 2018; Tao et al., 2018).

Its pathogenicity is attributed to the production of up to four different toxins (Osorio et al., 2018), and two main categories of strains can be distinguished. On the one side, strains harboring the virulence plasmid pPHDD1 produce the plasmid-encoded toxins damselysin (Dly) and phobalysin P (PhlyP) (Rivas et al., 2011), in addition to the chromosome I-encoded toxins phobalysin C (PhlyC) and the phospholipase PlpV (Vences et al., 2017). On the other side, strains lacking pPHDD1 only produce PhlyC and PlpV. Dly is a phospholipase-D active against sphingomyelin (Kreger et al., 1987) and PlpV is believed to be a phospholipase-A2 (Osorio et al., 2018), whereas PhlyP and PhlyC are pore-forming toxins (Rivas et al., 2015b). These four toxins are secreted via the type II secretion system (Rivas et al., 2015a; Vences et al., 2017). The highest virulence for fish is believed to be due to the additive functions of PhlyP plus PhlyC, and to the synergistic effect that both Dly and PlpV exert with the pore-forming toxins PhlyP and PhlyC (Rivas et al., 2013, 2015b; Vences et al., 2017). Strains with pPHDD1 exhibit wide hemolytic haloes on sheep blood agar plates whereas plasmidless strains cause narrow hemolytic haloes, and the two types of strains can be distinguished by this phenotypical test. Experimental inoculations have clearly demonstrated that strains harboring pPHDD1 are more virulent than plasmidless strains (Terceti et al., 2016; Vences et al., 2017). Studies conducted before the discovery of pPHDD1 had already suggested that fish farm outbreaks could be caused by the two types of strains (with wide and narrow hemolytic haloes, respectively) coexisting in the fish samples (Pedersen et al., 2009; Labella et al., 2010). Later studies proved that pPHDD1 occurs only within a fraction of *P. damselae* subsp. *damselae* strains (Rivas et al., 2014). A recent study has revealed that the *P. damselae* subsp. *damselae* populations which caused outbreaks in sea bass fish farms in the Turkish Black Sea lacked this plasmid, and it was also demonstrated that they constituted a multiclonal population with high genetic diversity (Terceti et al., 2016).

During the summer seasons of 1994 and 1995, *P. damselae* subsp. *damselae* was isolated for the first time as causative agent of outbreaks in rainbow trout fish farms, in Denmark (Pedersen et al., 1997). The outbreaks were coincident with periods of water temperatures up to 5°C higher than normal, from the beginning of July until mid-August. A few years later, in 2006, the Danish rainbow trout fish farms were again the scenario of *P. damselae* subsp. *damselae* outbreaks during an unusually warm summer season (Pedersen et al., 2008, 2009). The epidemiological analyses of strains from these 3 years uncovered a high genetic heterogeneity. Among six strains from 1994, three distinct ribotype patterns were identified, and the nine strains from 1995 yielded four distinct ribotypes, which were in turn different from those of the 1994 outbreaks (Pedersen et al., 1997). Notably, the analysis of 16 strains from the 2006 outbreaks revealed that each strain had a distinct PFGE pattern (Pedersen et al., 2009), providing sound evidence of a high genetic heterogeneity in the populations causing the outbreaks. These previous studies had not analyzed the distribution of virulence-related genes, since genetic diversity was evaluated by DNA-fingerprinting techniques. Nevertheless, the hemolytic phenotypes clearly differentiated a group of strains with strong hemolytic activity from a group of strains with weak hemolytic activity, and it was found that strongly hemolytic strains were 10,000 times more virulent (differences in four logarithmic units in the LD_50_) than the weakly hemolytic strains (Pedersen et al., 2009) (**Table 1**). Isolates from the rainbow trout outbreaks constitute a fantastic biological sample for analysis of genetic diversity in this pathogen, since they all come from the same fish host and the same area. In the present study, we have undertaken an in-depth genetic study of these 31 strains, and found evidence that different *P. damselae* subsp. *damselae* genotypes coexisted at the same time causing the outbreaks. Analysis of the whole genome sequences of four selected strains revealed a massive genetic heterogeneity. A number of mobile elements including pPHDD1 plasmid, putative prophages, as well as other virulence-related gene clusters and CRISPR-Cas systems showed a differential presence among isolates. From these results it is concluded that *P. damselae* subsp. *damselae* outbreaks can be caused by multiclonal populations rather than by specialized clonal lineages, and horizontal gene transfer has played a major role in shaping the genetic diversity within this subspecies.

**Table 1:** *Photobacterium damselae* subsp. *damselae* strains used in this study, isolated from head kidney of rainbow trout (*Oncorhynchus mykiss*) in Denmark.

Strain (short code)	Strain (original code)	Farm	Year of isolation	LD_50_^∗^	pPHDD1 plasmid ^§^	Phobalysin C (*hlyA_ch_* gene)	Sucrose phenotype on TCBS ^¥^	Collagenase (*colP* gene)	Reference
DK2	940804-1/1	A	1994		-	+	G	-	Pedersen et al., 1997
DK3	940804-1/2	A	1994		+	+	G	-	Pedersen et al., 1997
DK4	940804-2/1a	B	1994		+	+	G	-	Pedersen et al., 1997
DK5	940804-2/3	B	1994		+	+	G	-	Pedersen et al., 1997
DK6	940804-2/4	B	1994		+	+	G	-	Pedersen et al., 1997
DK7	940804-2/5a	B	1994		+	+	G	-	Pedersen et al., 1997
DK8	950810-3/2	C	1995		-	+	G	-	Pedersen et al., 1997
DK9	950810-3/4	C	1995		-	+	G	-	Pedersen et al., 1997
DK10	950810-3/5	C	1995		+	+	G	-	Pedersen et al., 1997
DK11	950823-1/3b	D	1995		-	+	G	+	Pedersen et al., 1997
DK12	950823-1/5	D	1995		-	+	G	+	Pedersen et al., 1997
DK13	950825-2/4a	E	1995		-	+	G	-	Pedersen et al., 1997
DK14	950828-1/3	D	1995		-	+	G	-	Pedersen et al., 1997
DK15	950901-2/2b	E	1995		-	+	G	+	Pedersen et al., 1997
DK16	950901-2/5b	E	1995		-	+	G	+	Pedersen et al., 1997
DK18	206308-4	N/A	2006	3.6 × 10^4^	+	+	G	-	Pedersen et al., 2009
DK19	206306-2	N/A	2006		+	+	G	-	Pedersen et al., 2009
DK20	206328-2	N/A	2006	3.9 × 10^3^	+	+	G	-	Pedersen et al., 2009
DK21	206306-6	N/A	2006		+	+	G	-	Pedersen et al., 2009
DK22	206328-5	N/A	2006		+	+	G	-	Pedersen et al., 2009
DK23	206303-14	N/A	2006		-	+	G	-	Pedersen et al., 2009
DK24	206302-7	N/A	2006		+	+	G	-	Pedersen et al., 2009
DK25	206320-5	N/A	2006		-	+	G	+	Pedersen et al., 2009
DK26	206276-1	N/A	2006	1.5 × 10^8^	-	-	G	+	Pedersen et al., 2009
DK27	206302-2	N/A	2006		-	-	G	+	Pedersen et al., 2009
DK28	206266-1	N/A	2006		-	-	G	+	Pedersen et al., 2009
DK29	206317-1	N/A	2006		+	+	G	+	Pedersen et al., 2009
DK30	206303-1	N/A	2006	1.5 × 10^7^	-	+	G	+	Pedersen et al., 2009
DK31	206308-1	N/A	2006		-	+	G	+	Pedersen et al., 2009
DK32	206352-6	N/A	2006		-	+	Y	-	Pedersen et al., 2009
DK33	206351-4	N/A	2006		-	+	G	-	Pedersen et al., 2009


*N/A, not available.*^∗^*LD_*50*_ for rainbow trout expressed in cfu, data from Pedersen et al. (2009).***^§^***pPHDD1 detection included PCR for dly and hlyA_pl_ genes and for pPHDD1 oriV.***^¥^***G, green colony; Y, yellow colony.*

## Materials and Methods

### Bacterial Strains and Culture Conditions

In two previous studies, a total of 31 *P. damselae* subsp. *damselae* strains were collected from head kidneys of diseased rainbow trout (*Oncorhynchus mykiss*) at several fish farms in Denmark (Pedersen et al., 1997, 2008). In 1994, six isolates were collected from six fish from two different farms; in 1995, nine isolates from nine fish from three different farms; and in 2006, 16 isolates from a total of seven different fish farms (**Table 1**). Strains were grown on tryptic soy agar or broth, supplemented with 1% NaCl (TSA-1 and TSB-1, respectively) and cultured at 25°C.

### Hemolysis and Motility Assays

Hemolysis assays on agar plates were conducted by picking a colony of each isolate previously grown on TSA-1, and inoculating it on sheep blood agar plates (Oxoid). For swimming motility assays, single isolated colonies of a 18 h culture agar plate for each strain were picked with a sterile plastic tip and stabbed into motility agar, which was prepared with TSB-1 broth supplemented with 0.25% bacteriological agar. For hemolysis and motility assays, pictures were taken at 24 h post-inoculation of the plates. Experiments were repeated three times to ensure that the hemolytic haloes and motility radius of the strains were reproducible.

### Assays for Phospholipase and Gelatinase Activities

The phospholipase/lecithinase activity was assayed using agar plates supplemented with egg yolk emulsion as a lecithin source. Ten microliters of TSB-1 overnight cultures for each *P. damselae* subsp. *damselae* strain were spotted onto TSA-1 plates supplemented with 3% egg yolk extract (Oxoid), and results were evaluated after 24 h of culture at 25°C. Hydrolysis of lecithin by the phospholipase yields water-insoluble diglycerides that cause the appearance of an opaque precipitate. The gelatinase activity assay was carried out by spotting 10 μl of a TSB-1 overnight culture onto TSA-1 plates supplemented with 1% gelatin (Oxoid), and results were developed after 48 h of incubation at 25°C by covering the agar plate surface with a 12.5% (wt/vol) HgCl_2_ solution. Hydrolysis of gelatin by the gelatinase enzyme causes the appearance of a translucent halo around the bacterial colony upon addition of HgCl_2_.

### Penicillin MIC Assay

To determine the susceptibility to penicillin, exponentially grown cultures of isolates DK2, DK3, DK20, and DK29 were adjusted to an OD_600_ of 0.5 and seeded onto TSA-1 plates in the presence of *E*-test gradient benzylpenicillin strips (bioMérieux).

### PCR

Relevant PCR primers used in this study are listed in **Supplementary Table S1**. PCR reactions were routinely performed with Kapa *Taq* DNA polymerase (Kapa) using a T-gradient thermocycler (Biometra). Routinely, the following thermal cycling conditions were used: 95°C for 5 min, followed by 30 cycles of 95°C for 30 s, 52.5°C for 30 s and an elongation step of 1 min at 72°C per kb.

### Molecular Phylogenetic Analysis

Evolutionary analyses were conducted in MEGA6 (Tamura et al., 2013). The evolutionary history of the strains was inferred using the Neighbor-Joining method (Saitou and Nei, 1987), and the analysis involved 31 *toxR* gene nucleotide sequences. The percentage of replicate trees in which the associated taxa clustered together in the bootstrap test (1,000 replicates) is shown next to the branches. The evolutionary distances were computed using the Maximum Composite Likelihood method (Tamura et al., 2004) and are in the units of the number of base substitutions per site.

### Genome Sequencing

Genomic DNA of strains DK2, DK3, DK20, and DK29 was purified using the GNOME DNA kit (Q-biogene), and sequenced using an Illumina MiSeq sequencer with 100× coverage. Reads were trimmed for quality, adapters and ambiguous nucleotides, and were assembled using SPAdes 3.6 (Nurk et al., 2013). Draft genome sequences were annotated and compared with the Rapid Annotations using Subsystems Technology (RAST Server) (Aziz et al., 2008). For the comparative analysis and the identification of common vs. specific genes among strains, putative orthologous genes were defined as reciprocal best hit proteins with a minimum 90% identity. Search of acquired antibiotic resistance genes (ARGs) was carried out through the four assembled genomes using the pipeline ResFinder (version 2.1) (Zankari et al., 2012) available at the Center for Genomic Epidemiology^1^. The threshold value for presence of an ARG was set to 50% similarity expressed as percent sequence identity (ID) and 60% of alignment length (coverage) of resistance gene. A total of 15 categories of ARGs were assayed, which included the following antimicrobials: Aminoglycosides; Beta-lactams; Colistin; Fluoroquinolones; Fosfomycin; Fusidic acid; Glycopeptides; MLS-Macrolide, Lincosamide and Streptogramin B; Nitroimidazole; Oxazolidinone; Phenicol, Rifampicin; Sulfonamide; Tetracycline; and Trimethoprim.

### Accession Numbers

DNA sequences have been deposited in GenBank database under accession numbers: PVXF00000000 (genome of strain DK2), PVXG00000000 (genome of strain DK3), PVXH00000000 (genome of strain DK20), and PVXI00000000 (genome of strain DK29).

## Results

### *A toxR* Gene-Based Phylogenetic Analysis Provides Evidence for a Multiclonal Origin of the *P. damselae* subsp. *damselae* Strains Associated With Rainbow Trout Outbreaks

In previous studies (Pedersen et al., 1997, 2008, 2009), *P. damselae* subsp. *damselae* was isolated as the causative agent of disease in marine rainbow trout farms in Denmark (**Table 1**). These studies revealed a lack of clonality among the strains, which exhibited a high diversity in their ribotype and PFGE patterns, suggesting that rainbow trout outbreaks were caused by genetically heterogeneous populations of *P. damselae* subsp. *damselae*. Albeit all the strains had been clearly assigned to *P. damselae* subsp. *damselae* by phenotypical tests in the aforementioned three previous studies, we wanted here to corroborate their taxonomic affiliation by testing for the presence of conserved gene markers. We proved that all the strains yielded positive amplification of the subspecies-specific *ureC* gene encoding a subunit of urease enzyme (Osorio et al., 2000), and all tested positive for the *rstAB* genes encoding a two-component regulatory system recently characterized in *P. damselae* subsp. *damselae* (Terceti et al., 2017) (data not shown).

**FIGURE 1:**
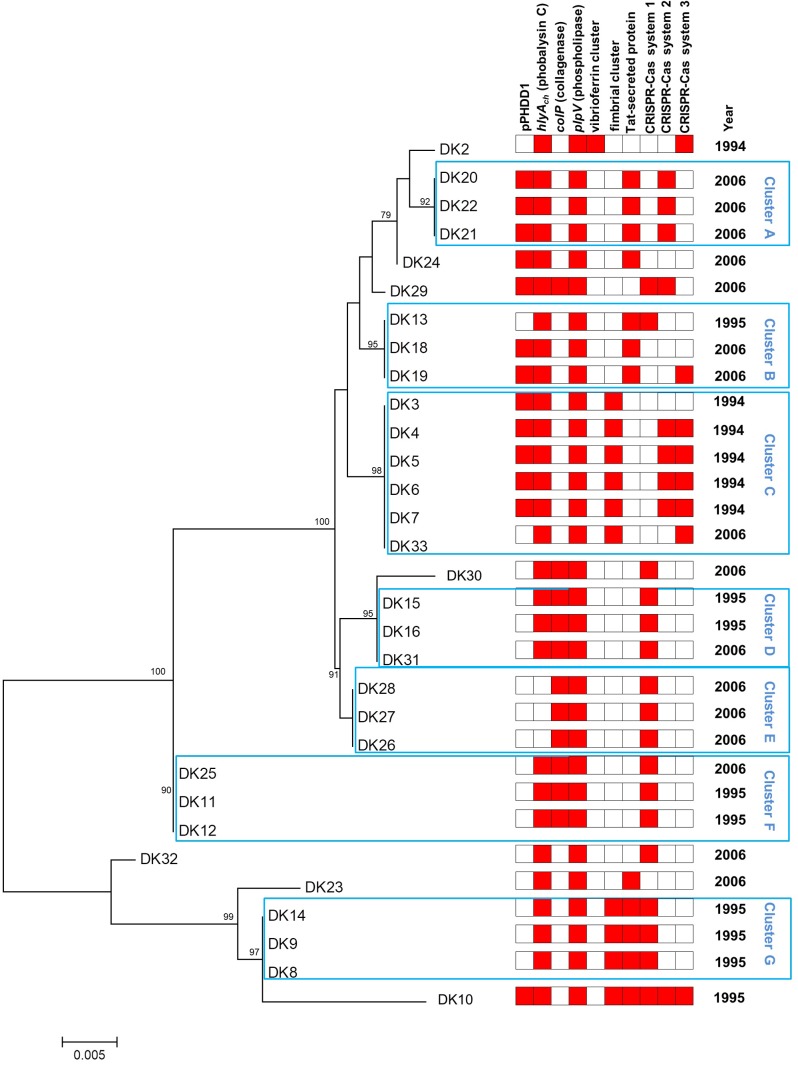
Phylogeny of 31 *P. damselae* subsp. *damselae* strains isolated from outbreaks in Danish rainbow trout farms. Neighbor-joining tree based on the alignment of complete *toxR* gene sequences of 31 strains. Numbers at the nodes indicate bootstrap values (% of 1,000 replicates; only bootstrap values of >70 are shown). The year of isolation (1994, 1995, or 2006) is also indicated. A heatmap illustration is shown to the right of the tree, and includes information regarding the presence of virulence plasmid pPHDD1, virulence genes *hlyA_ch_*, *colP*, and *plpV*, fimbrial gene cluster, twin-arginine (Tat) pathway protein, and three distinct CRISPR-Cas systems, 1, 2, and 3, respectively.

To demonstrate the hypothesis of the multiclonal origin of the Danish rainbow trout strains, here we PCR-amplified and sequenced the complete *toxR* gene in the 31 strains, and carried out a phylogenetic analysis. The *toxR* gene, which encodes a transmembrane transcriptional regulator of virulence genes, is considered a highly valuable molecular clock for fine-tuned discrimination of taxa within the *Vibrionaceae* due to its high variability (Osorio and Klose, 2000). As a result of the *toxR*-based analysis, we found that the 1994 outbreaks were caused by at least two different clones of *P. damselae* subsp. *damselae* (**Figure 1**), represented by strain DK2 on the one side, and DK3 to DK7 on the other side, respectively. Since all fish examined within an outbreak were received and sampled at the same time, the isolation of five strains (DK3-7) with identical *toxR* sequences likely indicates that at the time of the outbreak one specific genotype proliferated and caused an acute mortality event in farm A and B.

The nine strains from the 1995 outbreaks (DK8-16) from three farms depict a completely different landscape, as they are distributed in as many as five clusters in the phylogenetic tree. Interestingly enough, the study by Pedersen et al. (1997) already differentiated these nine strains into four biotypes, and there is almost a perfect correlation with those biotypes and the clusters determined in the present study: strains DK8, 9, and 14 (cluster G in **Figure 1**) correspond exactly to biotype 5 by Pedersen et al. (1997); DK11 and DK12 (cluster F) are biotype 7; DK15 and DK16 (cluster D) correspond to biotype 8; and, finally, strains DK10 and DK13 (biotype 6 in Pedersen et al., 1997) constitute an exception to the rule as they are distantly located in the *toxR* tree. Interestingly, the 1995 outbreaks also reveal that they were caused by multiclonal populations of *P. damselae* subsp. *damselae.* As an example, strains DK8–DK9 and DK10, isolated from the same farm, have very different *toxR* sequences and also different gene content (**Table 1** and **Figure 1**).

The 2006 outbreaks are represented by 16 strains collected from seven different fish farms, and these strains are distributed along almost all the clusters in the *toxR*-based phylogenetic tree. The first conclusion that can be drawn from the analysis of the 2006 outbreaks is their highly multiclonal nature. Some groups of strains seem to belong to the same genotype, as is the case of DK26-28 and DK20-22. The tree also reveals that some *toxR* genotypes from 2006 are identical to genotypes previously isolated in 1994 and 1995. However, none of the clones causing outbreaks in 1994 and 1995 became predominant enough as to displace other genotypes, and the 2006 outbreaks were indeed the most genetically diverse. It is also noteworthy that the majority of the clusters in the phylogenetic tree include strains from different outbreaks.

### *P. damselae* subsp. *damselae* Strains Contain Different Virulence Gene Repertoires

**FIGURE 2:**
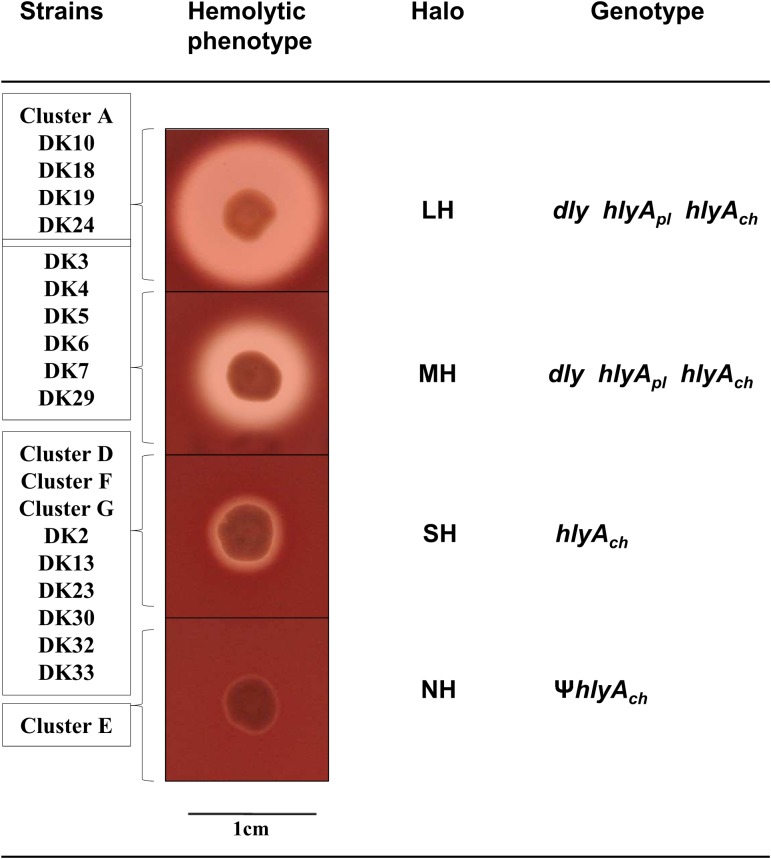
Representation of the four different categories of hemolytic phenotypes on sheep blood agar plates exhibited by the *P. damselae* subsp. *damselae* strains analyzed in this study: Large halo (LH), medium halo (MH), small halo (SH), and no hemolytic halo (NH). The individual strains and the complete clusters of strains belonging to each hemolytic category are listed at the left side of the pictures. The genotype of each hemolytic category is also detailed. The symbol Ψ denotes pseudogene.

Currently we know that *P. damselae* subsp. *damselae* can produce a number of virulence factors to cause pathogenicity in hosts. The four main virulence factors recognized so far have cytotoxic activity for different cell types (Osorio et al., 2018). Here, we found that the 31 rainbow trout strains could be divided into four distinct categories according to their haloes of β-hemolysis on sheep blood agar: a large β-hemolytic halo (LH) (7 strains), a medium halo (MH) (6 strains), a small halo (SH) (15 strains) or virtually absence of β-hemolytic halo (NH) (3 strains) (**Figure 2**). In order to ascertain the hemolysin gene content for each type of strain, we PCR tested for presence of each of the three major hemolysins, Dly, PhlyP and PhlyC, the ones that contribute to detectable phenotypes on sheep blood agar. Results demonstrated that 13 out of 31 strains (**Table 1**) tested positive for the three genes encoding Dly (*dly* gene), PhlyP (*hlyA_pl_* gene), and PhlyC (*hlyA_ch_* gene) hemolysins, and yielded also positive amplification of the pPHDD1 replication origin. These 13 strains, which correspond to the LH and MH strains, thus harbor a pPHDD1-like plasmid (**Figures 1**, **2**).

**FIGURE 3:**
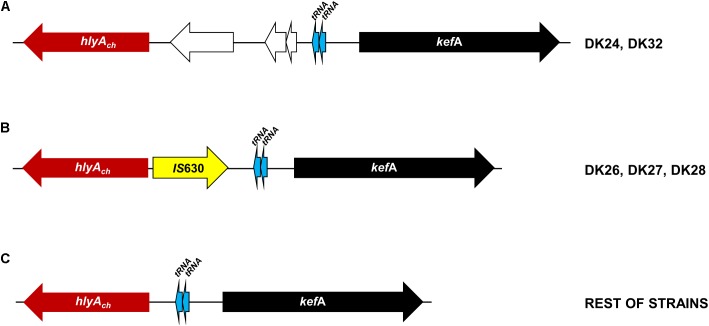
Scheme of the variable genomic regions upstream the *hlyA_ch_* gene encoding phobalysin C toxin in *P. damselae* subsp. *damselae* strains isolated from rainbow trout. The conserved *kefA* gene is represented as a black arrow, and two conserved tRNA genes are depicted as blue arrows. **(A)** Two strains DK24 and DK32 contain three extra genes (white arrows) located between a functional *hlyA_ch_* gene and the two tRNA genes. **(B)** The three non-hemolytic strains DK26, DK27, and DK28 contain an *IS*630-family transposase gene inserted within the *hlyA_ch_* promoter region, abolishing gene transcription and causing the loss of the hemolytic activity. **(C)** The remaining 28 isolates all are hemolytic and harbor a functional *hlyA_ch_* gene, and there is no additional DNA between *hlyA_ch_* and the two tRNA genes.

The totality of the 15 strains with small hemolytic halo (SH) tested positive for *hlyA_ch_* gene exclusively, and were negative for pPHDD1 replication origin. These strains will be here referred to as “plasmidless” strains. The three non-hemolytic (NH) strains, DK26, DK27, and DK28, tested negative for the complete *hlyA_ch_* gene but yielded partial amplification products of this gene instead, suggesting the presence of *hlyA_ch_* pseudogenes. To further examine this possibility, we conducted a PCR amplification and sequencing of the region flanking *hlyA_ch_* in the 31 *P. damselae* subsp. *damselae* strains. As a result, we found that the NH strains contained an *IS*630-family element inserted within the *hlyA_ch_* promoter sequence (**Figure 3**). This *IS*630 element was inserted at the same base pair (position 153 upstream the ATG start codon of *hlyA_ch_*) in the three strains, suggesting that they represent clonal colonies. These three strains also have identical *toxR* gene sequences (**Figure 1**).

**FIGURE 4:**
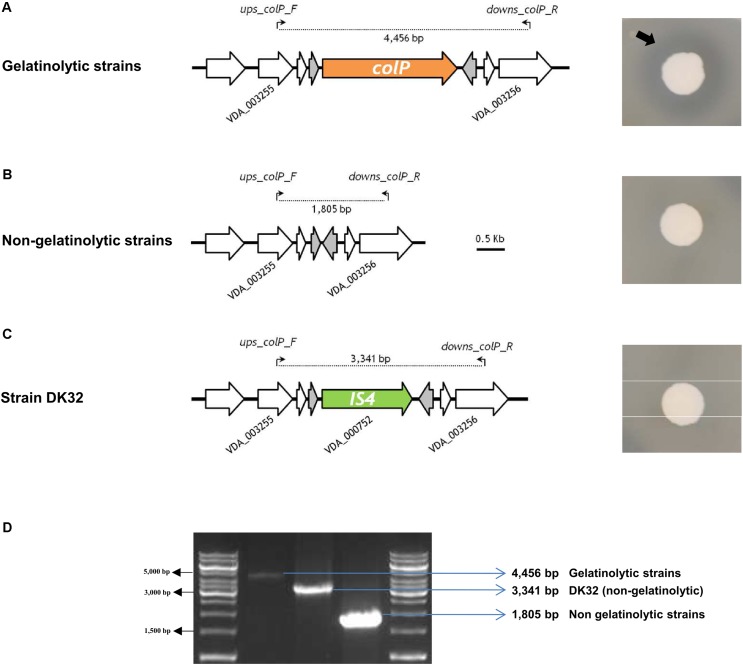
Gelatine-degrading activity of *P. damselae* subsp. *damselae* correlates with presence of *colP* gene encoding a collagenase within a variable DNA region. Colony pictures at the right side of the panel depict gelatinase activity detection on agar plates. A black arrow points at the border of the translucent gelatine degradation halo. **(A)** Strains with capacity to degrade gelatine all test positive for internal primers for *colP* gene (data not shown), which is located in the same conserved genome position in all the isolates. A PCR using the primer pair *ups-colp-F* and *downs-colp-R*, designed within the flanking genes VDA_003255 and VDA_003256, respectively, produces an amplicon of 4,456 bp. **(B)** Gelatinase-negative strains test negative for internal primers of *colP* gene (data not shown), and yield a smaller amplification fragment of 1,805 bp when tested with *ups-colp-F* and *downs-colp-R* primer pair. **(C)** The *colP*-negative strain DK32 yields a different amplicon band due to the insertion of a transposase gene between VDA_003255 and VDA_003256. **(D)** Agarose gel electrophoresis of the three different amplicon sizes produced with primer pair *ups-colp-F* and *downs-colp-R*, revealing three different genotypes in the variable region encoding ColP collagenase.

Since the fourth *P. damselae* subsp. *damselae* toxin, the phospholipase-A2 PlpV, does not produce detectable haloes on sheep blood agar by itself (Vences et al., 2017), we carried out a lecithinase agar test to gain evidence of the production of PlpV. As a result, we found that 13 strains yielded wide haloes, and these were coincident with the strains that tested positive for Dly and for the additional pPHDD1 gene markers, indicating that, as reported in a recent study (Vences et al., 2017), Dly phospholipase is a major contributor to lecithin degradation in this subspecies. The remaining 18 strains produced small lecithinase haloes (**Supplementary Figure S1**). The 31 strains tested positive for presence of *plpV* gene (**Figure 1**). These results are in agreement with the current knowledge that small haloes are caused by PlpV alone, whereas large haloes are the result of the combined lecithinase activities of Dly plus PlpV (Vences et al., 2017). Recently, a collagenase gene *colP* was reported to provide *P. damselae* subsp. *damselae* strains with the ability of degrading gelatin and collagen, and was shown to play a minor role in virulence (Vences et al., 2017). Using a PCR test specific for this gene, we here found that *colP* tested positive in 20 rainbow trout strains (**Table 1** and **Figure 1**), which also proved to be positive in a gelatinase agar plate assay. The remaining 11 strains tested negative for *colP* and were also negative for gelatin degradation on plate assays (**Figure 4**). A PCR analysis of the genetic context upstream and downstream *colP* gene revealed a conserved gene content in all the strains, with the exception of DK32 that contained an insertion sequence instead of *colP* gene, without disrupting any of the flanking genes (**Figure 4**). The intergenic region where *colP* is inserted overlaps with the transcriptional terminators of the two flanking genes. This observation, together with the finding of a clean insertion of an *IS* element in DK32, suggests that this genomic spot is prone to DNA acquisition.

### Additional Phenotypic Tests Also Reveal Heterogeneity of the *P. damselae* subsp. *damselae* Strains

As shown above, pPHDD1 plasmid and *colP* genes exhibit differential presence even within strains isolated from the same fish farm within an outbreak. We conducted additional phenotypical tests, which included the ability to degrade sucrose on TCBS agar. Only one strain (DK32) produced yellow colonies on TCBS, with the remaining 30 strains growing as green colonies (**Table 1**). Of interest was also the heterogeneity observed in the swimming motility phenotypes. Two strains were non-motile (DK23 and DK31), and the rest exhibited different levels of swimming motility. For the majority of the strains, it was observed that those with identical *toxR* sequences also exhibited a similar motility phenotype (**Supplementary Figure S2**).

### Complete Genome Sequencing of Four Rainbow Trout Strains Uncovers a High Number of Strain-Specific Genes, Potential Virulence Factors, and Mobile Elements

To gain an insight into the genomic divergences among strains, we obtained the draft genome sequences of DK2 and DK3 (from a 1994 outbreak in the same farm), DK20 and DK29 (from two different outbreaks in 2006). The general features of the four genomes are shown in **Table 2**. The genome size values and the %GC were similar to those reported for other strains of this subspecies (Vences et al., 2017). The core genome of the four genomes was established in 3,493 genes. Most notably, each strain proved to contain a large number of specific genes, which ranged from the 330 genes unique to DK29 to the 104 genes unique to DK20 (**Table 2**). DK2 lacked pPHDD1 plasmid, and the four genomes lacked the large plasmid pPHDD203 encoding a type III secretion system (T3SS), which has been previously reported only in the type strain so far (CIP 102761, GenBank Acc. No. ADBS00000000). Among the strain-specific genes, special attention was paid to DNA sequences encoding functions related to potential mobile DNA, including prophages, plasmids, and others (**Table 3**).

**Table 2:** General features of the four *Photobacterium damselae* subsp. *damselae* genomes analyzed in this study.

Characteristics	DK2	DK3	DK20	DK29
**Genome features**				
Genome size (bp)	4,360,322	4,494,653	4,479,365	4,616,326
GC %	40.53	40.51	40.51	40.47
Contig number	116	123	119	187
Genes total	3,874	4,023	3,992	4,175
CDS total	3,740	3,872	3,842	3,986
Number of unique CDS	154	140	104	330
**Virulence factors and other genes**				
pPHDD1 plasmid	-	+	+	+
T3SS (pPHDD203)	-	-	-	-
*hlyA_ch_* gene	+	+	+	+
*plpV* gene	+	+	+	+
*colP* gene	-	-	-	+
Vibrioferrin gene cluster	+	-	-	-
CRISPR-Cas systems	1	0	1	2


*Some virulence-related features are also highlighted.*

**Table 3:** A selection of strain-specific DNA regions deduced from the comparative genome analysis of the *P. damselae* subsp. *damselae* strains DK2, DK3, DK20, and DK29, with special emphasis on sequences related to horizontally acquired DNA, potential mobile elements, phage-related and plasmid-related proteins.

Strain	Contig n°	GenBank Acc. n°	Size in bp	Total ORFs in contig	Specific ORFs	General features of the strain-specific proteins
DK2	Contig_000002	PVXF01000002.1	2,598	5	3	Phage DNA packaging proteins; hypothetical proteins
	Contig_000003	PVXF01000003.1	2,221	3	3	Phage attachment proteins; VSK receptor, phage related protein; hypothetical protein
	Contig_000006	PVXF01000006.1	3,616	6	6	Plasmid-related partitioning protein ParA; phage proteins; hypothetical proteins
	Contig_000007	PVXF01000007.1	6,884	9	7	Phage proteins; phage terminase; hypothetical proteins
	Contig_000028	PVXF01000028.1	2,091	1	1	Phage protein
	Contig_000036	PVXF01000036.1	3,692	3	2	Phage proteins; hypothetical proteins
	Contig_000047	PVXF01000047.1	29,856	22	22	Siderophore vibrioferrin synthesis and transport; phage integrase; mobile element-related proteins; hypothetical proteins
	Contig_000051	PVXF01000051.1	1,767	1	1	Replication protein RepA
	Contig_000070	PVXF01000070.1	3,201	6	5	Phage replication initiation protein; hypothetical proteins
	Contig_000095	PVXF01000095.1	4,345	8	8	Phage anti-termination functions; exodeoxyribonuclease VIII; adenine-specific methyltransferase; hypothetical proteins
	Contig_000104	PVXF01000104.1	142,459	128	7	Response regulator; putative transcriptional regulator; hypothetical protein
DK3	Contig_000023	PVXG01000023.1	2,772	3	2	Phage tail length tape-measure protein; hypothetical protein
	Contig_000037	PVXG01000037.1	122,163	101	8	Fimbrial protein precursor; chaperone protein; outer membrane usher protein; hypothetical proteins
	Contig_000038	PVXG01000038.1	133,769	125	3	Phage T7 exclusion protein; hypothetical proteins
	Contig_000042	PVXG01000042.1	188,886	19	4	Phage integrase; hypothetical proteins
	Contig_000043	PVXG01000043.1	47,455	47	4	Bacteriophage f237 ORF9; hypothetical protein
DK20	Contig_000020	PVXH01000020.1	4,907	3	2	Plasmid-related proteins
	Contig_000039	PVXH01000039.1	120,633	103	9	Type I restriction-modification system; DNA replication helicase; hypothetical proteins
	Contig_000070	PVXH01000070.1	16,594	14	4	Phage proteins; hypothetical proteins
DK29	Contig_000011	PVXI01000011.1	8,573	6	6	CRISPR-associated proteins (Cas), type I-F
	Contig_000012	PVXI01000012.1	23,216	24	24	Recombinational DNA repair protein RecT (prophage associated) putative primase; hypothetical proteins
	Contig_000046	PVXI01000046.1	3,771	1	1	Integrase
	Contig_000055	PVXI01000055.1	32,765	22	18	IncF plasmid conjugative transfer pilus proteins TraN, TraU, TraW; hypothetical proteins
	Contig_000071	PVXI01000071.1	114,282	110	49	Phage tail fiber proteins, phage replication and other phage-related proteins; hypothetical proteins
	Contig_000106	PVXI01000106.1	36,123	34	25	Phage proteins; hypothetical proteins; replication initiation protein RepE
	Contig_000116	PVXI01000116.1	39,314	38	32	Site-specific recombinase, phage integrase family; transposase; hypothetical proteins
	Contig_000127	PVXI01000127.1	5,101	8	3	Cyanophage-encoded porphyrin biosynthetic protein; hypothetical proteins
	Contig_000151	PVXI01000151.1	47,345	44	36	Plasmid conjugative transfer pilus assembly proteins TraB, TraC, TraD, TraE, TraF, TraH, TraK; hypothetical proteins


The genome sequence of DK2 revealed a total of 154 unique genes, all absent in the other three sequenced genomes. Most of these genes encoded putative hypothetical proteins, but some of them were annotated as putative phage proteins and restriction-modification systems. Notably, a contig of 29,856 bp unique to this strain was found to include a group of genes predicted to participate in the synthesis and uptake of the siderophore vibrioferrin (**Table 3**), a siderophore originally identified in *Vibrio parahaemolyticus* (Yamamoto et al., 1994). Indeed, recent studies have shown that some strains of *P. damselae* subsp. *damselae* produced this siderophore and used it as iron scavenger (Balado et al., 2017; Puentes et al., 2017). Vibrioferrin production showed to be a variable trait in the subspecies, and a recent study has uncovered that many virulent strains do not produce this siderophore and test negative for *pvs* genes (Balado et al., 2017). We therefore designed two different primer pairs, targeted to the biosynthetic genes *pvsB* and *psvD*, respectively, and assayed the presence of these genes among the 31 rainbow trout strains. We found that these genes were exclusively found in DK2 (**Figure 1**). The observation that the contig containing the vibrioferrin gene cluster also contains a number of insertion sequences and other mobile element-related genes (**Table 3**), suggests that it might have been acquired by horizontal gene transfer by some *P. damselae* subsp. *damselae* lineages.

Strain DK3 harbors 140 unique genes, including many phage-related proteins (**Table 3**). An interesting feature of this strain is the existence of a putative fimbrial operon of five genes that proved to be absent in the other three genomes (see below).

The genome sequence of DK20 showed 104 specific genes, the lowest number among the four genomes analyzed. Most of them accounted for hypothetical proteins, mobile-element related functions and phage proteins (**Table 3**).

Strain DK29 contained the largest number of specific genes, which accounted for a total of 330. Not surprisingly, this strain has the largest genome of the four analyzed (**Table 2**). The majority of the unique genes were found to be clustered in several large contigs (**Table 3**). One contig contained a CRISPR-Cas system of the type I-F (see below). Five contigs contained phage-related genes. Two contigs which accounted for ca. 80 kb of DNA unique to DK29 contained features of plasmid DNA including an IncF-type conjugative system, suggesting that they correspond to one or more putative novel plasmids as these DNA sequences showed little similarity to known sequences (data not shown).

#### CRISPR-Cas Systems

**FIGURE 5:**
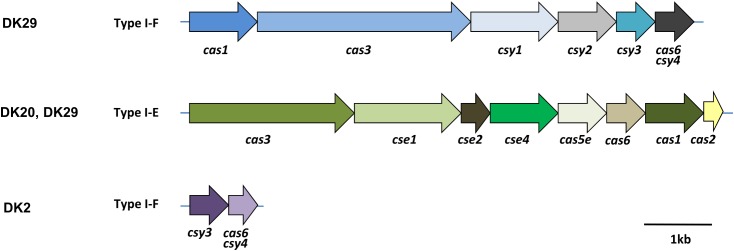
Architectures of the genomic loci encoding predicted Cas proteins of CRISPR-Cas systems in the genomes of the *P. damselae* subsp. *damselae* isolates DK2, DK20, and DK29. The locus tag names are indicated, following the classification from Makarova et al. (2015).

Identification of CRISPR-Cas systems in *P. damselae* subsp. *damselae* has been neglected in previous studies. The sequencing of four genomes in the present study uncovered several clusters encoding predicted Cas proteins (**Figure 5**). Strain DK29 contained two different CRISPR-Cas systems of types I-F and I-E, respectively, with the typical signature protein Cas3. The DK20 genome also encoded a I-E type system, virtually identical to that encoded by DK29 genome. Strain DK2 encoded two Cas proteins of a putative type I-F system. No Cas proteins could be deduced from the genome annotation of DK3. In order to gain an insight into the distribution of each of these three CRISPR-Cas systems among the *P. damselae* subsp. *damselae* collection, we designed tailored primer pairs to screen for a number of signature genes. The results uncovered a high genetic heterogeneity, with strains harboring one Cas protein clusters, other isolates harboring the three of them, and two strains testing negative for the three assayed Cas systems (**Figure 1**).

#### Antibiotic Resistance Genes

The four sequenced genomes possessed two conserved genes encoding putative beta-lactamases, which is in agreement with the resistance to penicillin observed in the four isolates (**Supplementary Figure S3**). These genes appear to be encoded on the chromosomes and not on plasmids, suggesting that they have been part of the *P. damselae* subsp. *damselae* genome for long. In a previous study (Pedersen et al., 2009), it was reported that strains DK20 and DK29 exhibited resistance to sulphonamides. However, the search of acquired ARGs using the pipeline ResFinder (version 2.1) (Zankari et al., 2012) and setting the threshold value for presence of an ARG to 50% similarity, yielded negative results for the four genomes sequenced in the present study. Visual inspection of annotation files produced by RAST server also failed to uncover additional ARGs. Moreover, none of the four genomes contained sequences with significant similarity to pAQU1, a resistance plasmid of 204 kb described in *P. damselae* subsp. *damselae* (Nonaka et al., 2012).

#### Additional Mobile Elements: Screening for a Plasmid-Encoded T3SS Yields Negative Results

The type strain of this subspecies (CIP 102761) is known to harbor a 203 kb-plasmid dubbed pPHDD203, encoding a T3SS and several putative effectors (Vences et al., 2017). We wanted to screen for the presence of this secretion system in the rainbow trout strains, and for such purpose we designed two primer combinations targeted to two genes of the T3SS apparatus: locus VDA000187, encoding inner membrane protein YscD and locus VDA000193, encoding an inner membrane channel protein, respectively. In addition, we designed a primer pair targeted to the locus VDA000224 that encodes a putative protein tyrosine phosphatase effector, homologous to AopH. As a result of this PCR screening we found that the 31 strains tested negative, and only the type strain, used here as a positive control, yielded amplification of the three T3SS gene markers (data not shown), providing strong evidence that the T3SS constitutes more the exception than the norm in *P. damselae* subsp. *damselae*. In support of this, three previously published *P. damselae* subsp. *damselae* genomes also proved to lack a T3SS (Vences et al., 2017).

### A Number of Chromosomal Regions Exhibit a High Degree of Genetic Plasticity Among *P. damselae* subsp. *damselae* Genomes

#### The Region Downstream the Conserved *trpR* Gene Is a Potential Hot Spot for DNA Acquisition in *P. damselae* subsp. *damselae* Genomes

**FIGURE 6:**
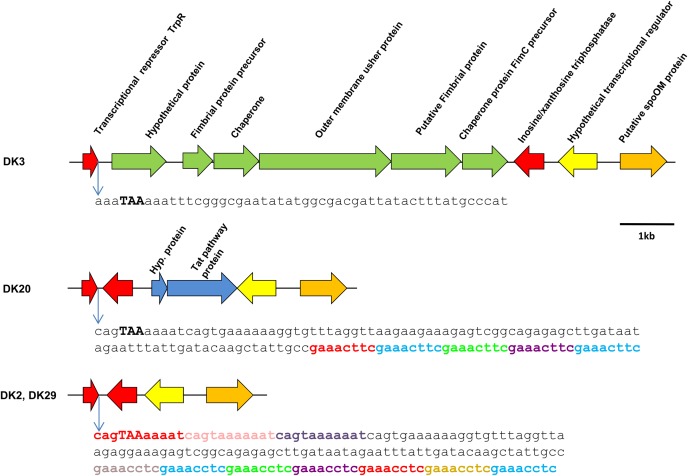
Scheme of the variable genomic region downstream the conserved gene *trpP* in *P. damselae* subsp. *damselae* genomes. The fimbrial gene cluster found in strain DK3 in this position, is also present in nine additional strains (**Figure 1**). A total of 12 strains, including DK20, contain a different gene repertoire that includes the genes of a hypothetical protein of 11 kDa and a putative protein secreted by the Twin-arginine (Tat) pathway, respectively. The genomes of DK2 and DK29 do not harbor genetic material inserted between the two conserved genes depicted in red. Interestingly, the putative transcriptional terminator region of *trpP* contains a series of 8-mer and 11-mer direct repeats (represented in different colors) that might play a role in foreign DNA acquisition. The stop codon of *trpR* is represented in high caps and bold. Note that the sequence downstream this stop codon differs between strains.

As mentioned above, strain DK3 was found to harbor a gene cluster coding for fimbrial proteins, chaperones and outer membrane usher proteins (**Figure 6**). A comparative analysis of this DNA region in the four genomes revealed as many as three distinct gene repertoires: DK2 and DK29 contained genes conserved in the four strains, DK3 contained the fimbrial operon, and DK20 contained two genes encoding an hypothetical protein and a protein of unknown function predicted to be secreted by the twin-arginine (Tat) pathway, respectively (**Figure 6**). These two proteins were found to be homologous to proteins previously identified in other *P. damselae* genomes. However, the closest homologs of the five fimbrial-related proteins belonged to species of the genus *Shewanella*, suggesting that this variable region has been acquired by horizontal gene transfer as a block from a *Shewanella*-related bacterium (**Supplementary Table S2**). Such genetic heterogeneity suggests that this genome region constitutes a hot-spot for recombination of foreign DNA. In support of this hypothesis, we found that the intergenic region between the conserved genes encoding for TrpR repressor and for an inosine-xanthosine triphosphatase, respectively, contained a number of tandem repeats of the 8-mer sequence GAAAC(C/T)TC in strains DK2, DK20, and DK29, and a three-repeat of the sequence CAGTAAAAAAT in strains DK2 and DK29. These repeats likely overlap with the putative transcriptional terminator downstream of *trpR* gene. Overall, the sequence immediately downstream the *trpP* stop codon shows heterogeneity among the studied strains (**Figure 6**).

We designed primer pairs to screen for the differential presence of these two blocks of variable DNA (the fimbrial gene cluster, and the Tat-pathway protein, respectively) among the 31 *P. damselae* subsp. *damselae* strains. As a result, we found that these two gene blocks exhibited a variable presence among the *P. damselae* subsp. *damselae* population. Six strains tested positive only for the fimbrial genes, eight strains contained only the Tat-pathway protein, thirteen strains were negative for the two clusters, and four tested positive for the two of them: DK8, DK9, DK14 (all in cluster G), and DK10. Interestingly, the strains belonging to the same cluster in the *toxR* phylogenetic tree, showed the same gene content in this variable region, supporting the strong value of *toxR* as a fine-tuned phylogenetic marker (**Figure 1**).

#### The Chromosome I Region Encoding PhlyC Hemolysin Contains Strain-Specific Gene Combinations: Evidences of Extensive DNA Recombination and Gene Acquisition by Horizontal Transfer

**FIGURE 7:**
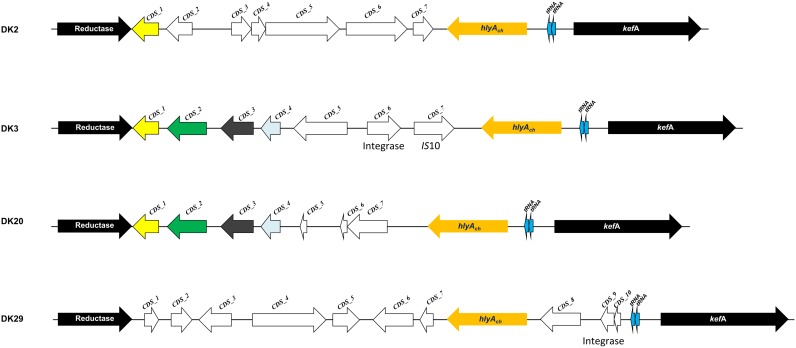
Scheme depicting the diversity of gene repertoires found in the genomic context of *hlyA_ch_* gene encoding phobalysin C toxin in *P. damselae* subsp. *damselae* genomes. Two conserved flanking genes are represented as black arrows (reductase gene and *kefA* gene). Arrows with the same color code are shared by more than one genome, and white arrows represent ORFs unique to a single genome. A total of 26 unique genes were found within this variable region among the four genomes. Sequence features related to acquisition of DNA by horizontal gene transfer, which include two tRNA genes, integrase genes and *IS*10 elements, are also indicated.

Phobalysin C toxin is encoded by *hlyA_ch_* gene located in chromosome I (Rivas et al., 2013). As shown above, 28 out of the 31 rainbow trout strains harbor a functional *hlyA_ch_* gene, whereas strains DK26-28 likely constitute clonal derivatives that have a disrupted *hlyA_ch_* gene due to the insertion of an *IS*630-family element. A previous study pointed out that the chromosome I region harboring *hlyA_ch_* gene might constitute a hot spot for recombination of horizontally acquired DNA (Rivas et al., 2014). Therefore, we examined the *hlyA_ch_* gene context in the four complete genomes obtained in the present study. Interestingly, we found that each strain contained a unique gene combination downstream of *hlyA_ch_* gene (**Figure 7**). Notably, among all the genes that constitute the variable fraction in the four strains (26 different genes in total), only six genes have been previously described in *P. damselae* subsp. *damselae* so far (**Supplementary Table S3**). The remaining ORFs showed similarity to different *Vibrio* and *Photobacterium* species, and two genes in DK29 were likely acquired as a block from a *Pseudoalteromonas flavipulchra*-related bacterium. The diverse taxonomy of the species showing closest homologs to genes within this variable region, clearly indicates their heterogeneous origin, and suggests that the genome region encoding PhlyC is subjected to frequent events of gene gain and loss and to extensive recombination with DNA sequences acquired from other marine bacteria. In support of this hypothesis, we could detect the presence of two genes encoding transfer RNAs within these variable regions, as well as two integrase genes and an *IS*10-family transposase gene, all of which are candidates to be involved in events of DNA acquisition (**Figure 7**).

#### Extracellular Polysaccharide Synthesis (EPS) Clusters Exhibit a High Variability and Contain Many Strain-Specific Genes

**FIGURE 8:**
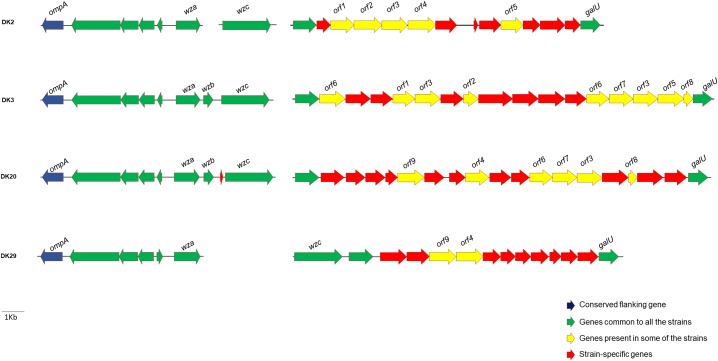
Diversity in gene content of the clusters encoding functions related to synthesis of cell envelope polysaccharides (LPS and capsular polysaccharides) in four *P. damselae* subsp. *damselae* genomes. Note that each strain contains a large number of unique genes, represented as red arrows.

There is evidence that *P. damselae* subsp. *damselae* is an antigenically diverse pathogen. Previous studies have analyzed a reduced number of strains, and reported as many as 7 O-serogroups among 16 strains analyzed (Fouz et al., 1992). A recent comparative analysis conducted with virulent strains of this pathogen, revealed that strains of different serogroups contain distinct gene repertories within a putative cluster for cell envelope polysaccharide synthesis (Osorio et al., 2018). We therefore searched the polysaccharide synthesis homologous clusters within the genomes of strains DK2, DK3, DK20, and DK29. To aid in the identification of DNA regions encoding the polysaccharide synthesis clusters, we searched for genes *wza*, *wzb* and *wzc*, homologs of well-studied *Escherichia coli* genes known to be involved in the synthesis of colanic acid and of capsules of groups 1 and 4 (Whitfield, 2006). We found that the four genomes shared a group of conserved genes which included *wza* and *wzc* among others (**Figure 8**). However, the region between *galU* (UTP-glucose-1-P uridylyltransferase) and *wzc* genes showed to contain a high number of strain-specific genes, suggesting that each *P. damselae* subsp. *damselae* strain synthesizes different polysaccharide molecules.

## Discussion

*Photobacterium damselae* subsp. *damselae* is recognized as an emerging pathogen in marine aquaculture, and outbreaks in fish farms have been correlated with episodes of unusually high temperatures (Pedersen et al., 1997, 2008, 2009; Terceti et al., 2016), following similar patterns as other infections caused by vibrios (Le Roux et al., 2015). We showed here that the *P. damselae* subsp. *damselae* strains from outbreaks during 3 years in rainbow trout farms in Denmark exhibited a high diversity of phenotypes and genotypes. This collection of strains had been previously characterized by DNA fingerprinting techniques (ribotyping and Pulsed-Field-Gel-Electrophoresis) in two studies (Pedersen et al., 1997, 2009), revealing a lack of clonality among the strains and suggesting that rainbow trout outbreaks were caused by genetically heterogeneous populations. In this same line of ideas, unrelated studies also reported the existence of high genetic heterogeneity among *P. damselae* subsp. *damselae* strains from the same geographical area (Botella et al., 2002; Terceti et al., 2017).

In the present study, we detected one strain (DK32) producing yellow colonies on TCBS. Most *P. damselae* subsp. *damselae* strains do not ferment sucrose and thus produce green colonies on TCBS medium (Fouz et al., 1992; Botella et al., 2002; Vaseeharan et al., 2007; Takahashi et al., 2008; Abdel-Aziz et al., 2013), but strains forming yellow colonies are isolated from time to time (Zhao et al., 2009; Terceti et al., 2016) and there is no known reason to consider sucrose-positive strains as atypical.

Recent studies have demonstrated the utility of *toxR*-based analyses for epidemiological studies of *P. damselae* subsp. *damselae*, since sequence polymorphisms in this gene allow the discrimination of closely related strains and can unveil the existence of different clones within an outbreak (Alba et al., 2016; Terceti et al., 2016). In the present study, we found an almost perfect correlation between groups of strains that clustered together in the *toxR*-based phylogenetic tree, and the clustering of the same strains into biotypes in previous studies (Pedersen et al., 1997, 2009). In addition, strains considered as different sequence types according to their *toxR* sequences, were also found to contain different gene repertoires (**Figure 1**).

The isolation of DK2 and DK3 from two different fish individuals, respectively, in farm A in 1994, indicates that fish had been colonized by at least two distinct *P. damselae* subsp. *damselae* genotypes. Interestingly, the *toxR*-based tree revealed that some genotypes from 2006 were identical to genotypes previously isolated in 1994 and 1995. This observation suggests that some *P. damselae* subsp. *damselae* genotypes might thrive in the environment during large periods of time between outbreaks, and cause fish outbreaks as soon as the environmental and/or host conditions are favorable. Still, the sampling procedure allowed the isolation of potentially clonal strains, exemplified by DK3 to DK7 from 1994. Interestingly enough, these clonal strains all harbor pPHDD1 plasmid. Previous studies have suggested that highly virulent (i.e., pPHDD1-harboring) strains have more chances to become temporarily clonal in a fish farm (Fouz et al., 1992). The *toxR*-based analysis, together with the screening of virulence-related genes, demonstrated that the *P. damselae* subsp. *damselae* populations causing outbreaks in rainbow trout farms are multiclonal in nature.

A recent study on comparative genomics pointed at the existence of a high degree of diversity within the *Photobacterium* genus (Machado and Gram, 2017), and the core genome for the genus was inferred to comprise 1,232 genes, after comparing a total of 35 *Photobacterium* genomes. In our study, we have found that a pair of strains (DK2 and DK3) isolated from the same outbreak at the same fish farm contained a high number of strain-specific genes. It is pertinent to say that the present study constitutes, to the best of our knowledge, the first report of comparative genomics analysis in the species *P. damselae* in which strains from the same outbreak are compared. A recent study analyzed the genome sequence of three strains from this subspecies, but the strains came from different host species and from different geographical locations (Vences et al., 2017). Our study has uncovered the differential occurrence of a number of virulence-related genes throughout the 31 strains from rainbow trout. Some virulence traits have been shown to be more the exception that the rule within the *P. damselae* subsp. *damselae* populations. This is the case of the plasmid-encoded T3SS previously identified in the type strain (Vences et al., 2017), that showed to be absent in all the Danish rainbow trout strains. Also, a putative gene cluster for the synthesis and utilization of siderophore vibrioferrin showed to be exclusive of strain DK2, supporting previous observations that this genetic system is only characteristic of a fraction of *P. damselae* subsp. *damselae* strains (Balado et al., 2017).

The four major virulence factors investigated so far in *P. damselae* subsp. *damselae* are cytotoxins with hemolytic activity (Osorio et al., 2018). Our study has confirmed that *dly* and *hlyA_pl_* genes, when present, always occur simultaneously, and their presence is linked to the detection of the replication origin of pPHDD1 plasmid. A reduced percentage of strains (3 out of 31) harbored a pseudogene of *hlyA_ch_* (encoding PhlyC), whereas the phospholipase-encoding *plpV* gene and its associated lecithinase activity proved to be ubiquitous in all the strains. These observations confirm that PlpV phospholipase is the unique ubiquitous virulence gene marker described in *P. damselae* subsp. *damselae* so far, as previously suggested (Vences et al., 2017). It is known that PlpV exerts some hemolytic activity against trout erythrocytes, and this activity is enhanced in the presence of lecithin (Vences et al., 2017).

We also found a perfect correlation between the hemolysin gene content, and the size of the hemolytic haloes reported by a previous study (Pedersen et al., 2009). It is pertinent to say that this previous study had clearly differentiated a group of strong hemolytic activity from a group of weak hemolytic activity, and found that a strongly hemolytic strain was 10,000 times more virulent than the weakly hemolytic strain (**Table 1**). Therefore, the virulence gene screening has demonstrated that the rainbow trout outbreaks were likely caused by a mixture of pPHDD1-negative and pPHDD1-containing strains, or, to say it with other words, by strains with very different virulence degrees. This observation may question whether the plasmidless strains were in fact causative organisms of the disease or whether they behaved as secondary invaders with a minor role in the infection. It is interesting to note that eight out of the nine strains from the 1995 outbreak were pPHDD1-negative (**Table 1**), and most of these strains were recovered from diseased fish that rendered pure cultures after sampling (Pedersen et al., 1997).

Horizontal gene transfer is a major driving force in bacterial evolution and diversification (Ochman et al., 2000; Thomas and Nielsen, 2005). Genomic variation among *P. damselae* subsp. *damselae* genomes analyzed here appeared to be associated with the differential occurrence of putative mobile DNA. This variable DNA fraction could have been gained by the contribution of each of the three main mechanisms of gene acquisition by horizontal gene transfer. Hence, the virulence plasmid pPHDD1 was likely acquired by conjugation, since a previous study demonstrated that this plasmid could be mobilized into recipient *P. damselae* cells (Rivas et al., 2011). The major role of phages in DNA acquisition is exemplified by the number of putative phage-associated genes that were found in the genomes of the four *P. damselae* subsp. *damselae* strains analyzed (**Table 3**). The comparative analysis of these four genomes has also pointed at the existence of several hot-spots for DNA acquisition. Some hypervariable regions contain short repetitive sequences in tandem which overlap with the putative transcriptional terminators of genes. A good example was provided here with the putative fimbrial gene cluster of strain DK3. Interestingly, some naturally transformable Gram-negative bacteria (e.g., *Haemophilus* spp. and *Neisseria* spp.) contain DNA uptake signal sequences within the base-paired stems of transcriptional terminators (Smith et al., 1999; Ambur et al., 2007; Spencer-Smith et al., 2016). These repeated sequences might therefore have played a role in the recombination of horizontally acquired DNA in some *P. damselae* subsp. *damselae* isolates. Additionally, a hypervariable region was characterized in the vicinity of the *hlyA_ch_* gene encoding PhlyC toxin. This region, in addition to integrase genes and *IS* elements, also contained several tRNA genes. The role of tRNA genes as target loci for the insertion of horizontally acquired DNA sequences has been extensively documented (Schmidt and Hensel, 2004; Castillo et al., 2017). The increasing evidence that *Vibrionaceae* species are indeed prone to gain foreign DNA by natural transformation (Sun et al., 2013; Antonova and Hammer, 2015; Metzger and Blokesch, 2016), suggests that some of these variable DNA sequences might have been taken from the environment and inserted into the *P. damselae* subsp. *damselae* genomes via the aforementioned repeated sequences. This hypothesis will surely deserve an in-depth investigation in the near future.

Of particular significance is the overwhelming diversity within the gene clusters encoding cell envelope polysaccharides (LPS and capsular polysaccharides). It is known from early studies that this pathogen exhibits serological diversity (Fouz et al., 1992). Our study provides supporting evidence that polysaccharide synthesis clusters of *P. damselae* subsp. *damselae* exhibit unique gene combinations in each analyzed strain, as previously suggested (Osorio et al., 2018). The reason for this massive genetic diversity is currently unknown, and it might be related to the lifestyle of this generalist pathogen, that is capable of infecting a wide range of animals and also of living as a free-swimming bacterium (Fouz et al., 1998, 2000).

This study has also brought into attention the abundance and diversity of CRISPR-Cas systems occurring in *P. damselae* subsp. *damselae* genomes. Genome sequencing of only four strains revealed as many as three distinct Cas gene clusters which, according to the recent classification of CRISPR-Cas (Makarova et al., 2015) likely belong to the types I-F and I-E. The PCR screening revealed that most of the 31 analyzed strains from rainbow trout outbreaks harbor at least one of these systems. As a pathogen that also thrives as a free-living bacterium in seawater, *P. damselae* subsp. *damselae* lives in close contact with bacteriophages (Novianty et al., 2014; Yamaki et al., 2015), hence the abundance of CRISPR-Cas systems. Not surprisingly, much of the variable DNA content that showed to be specific of each of the four genomes analyzed here contained typical features of prophage DNA. Recently, it has been reported that CRISPR-Cas systems may also play a regulatory role on endogenous genes. Thus, in addition to protecting from phages, these systems are increasingly being recognized as regulators of bacterial virulence (Louwen et al., 2014; Ma et al., 2018).

## Conclusion

The results presented here have provided an in-depth picture of the epidemiology of the outbreaks caused by *P. damselae* subsp. *damselae* in Danish rainbow trout farms in 1994, 1995, and 2006. An overview of the virulence gene repertoires plus the presence of additional markers has revealed a high degree of genetic variability within this subspecies. In addition, as illustrated in **Figure 1**, it can be concluded that *P. damselae* subsp. *damselae* outbreaks are caused by multiclonal populations. High-virulence (presence of pPHDD1 plasmid) and low-virulence (absence of pPHDD1) strains coexist within an outbreak. Therefore, further research is needed in order to clarify whether pPHDD1-negative strains are in fact causative organisms of the disease, or whether they play a secondary role in infection. The variability of polysaccharide biosynthesis genes and other gene markers among strains is overwhelming, and horizontal gene transfer is believed to have played a major role in the diversification of this subspecies, since much of the strain-specific DNA had features related to plasmids, prophages and pathogenicity islands. *P. damselae* subsp. *damselae* is a fascinating microorganism, with a high genetic diversity, and constitutes a very good model for studying the role of horizontal gene transfer as a driving force in the evolution of bacterial pathogens.

## Author Contributions

MT and CO performed the experiments and wrote the manuscript. MT, AV, XM, and CO performed analysis and interpreted the results. KP and ID provided the strain collection and significantly contributed to data interpretation. CO designed the study and directed the research. All the authors read and approved the final manuscript.

## Conflict of Interest Statement

The authors declare that the research was conducted in the absence of any commercial or financial relationships that could be construed as a potential conflict of interest.

## Footnotes

1 http://cge.cbs.dtu.dk/services/
