# Supplementary material for: Molecular Epidemiology of Photobacterium damselae subsp. damselae Outbreaks in Marine Rainbow Trout Farms Reveals Extensive Horizontal Gene Transfer and High Genetic Diversity
Source: Front Microbiol. 2018 Sep 19;9:2155. doi: 10.3389/fmicb.2018.02155 (PMC6156455; doi:10.3389/fmicb.2018.02155)
Supplement: Supplementary file 1 [file Table_1.PDF]

Supplementary Table S1. Oligonucleotides used in this study.

Target and oligonucleotides	Sequence	bp amplified
dly		
Dly-5’	CTCCTATGGGACATGAATGG	549
Dly-3’	TGCTCTAGGCTAAATGAATC	
phlyP (hlyA$_{pl}$)		
PhlyP-5’	GCTATAAATGAATAAGAAAA	767
PhlyP-3’	TTGAAGCTAACTCAAAAA	
phlyC (hlyA$_{ch}$)		
phlyC-5’	AATGTTTCTTTCCGTTGGGC	353
phlyC -3’	CCGGAGTTCCACCAGTAAAT	
hlyAch-det(hlyA$_{ch}$)		
hlyAch-det-5’	GAAGAGTTAGCGATGGTCTA	288
hyAch-det-3’	TTGATATAGCGTCTGCGCCC	
hyAch (upstream sequences)		
hlyAch-3new	ACATATACAATACGATCTGG	variable
KefA-3’	TGTAGGTAGATATCGAGCTC	
PlpV int (phospholipase)		
plp int -F	TCTCATAATAGCAGTAATCT	1618
plp int -R	TTACTAAGCAGAATCCAGCC	
pPHDD1 replication origin		
Ori_pPHDD1-F	TGGAATAACTATGAGTAACA	3052
Ori_pPHDD1-R	CATTACCAAAACATCTACAT	
toxR		
toxR-5’	GGGATTTTATGGTACACAAA	985
toxR-3’	ATCATAACCAGAGAGATGCT	
Collagenase (internal fragment)		
col - F	CCTGCTACAGTTCGAGCTCA	1617
col - R	TCAACCACTCGAGATATTCC	
Collagenase (gene context)		
ups_colP_F	GAGGACCATTTATTGCCAGT	variable
downs_colP_R	CGGCTAAGCAAACAGAAAAC	
Urease		
Ure-5’	TCCGGAATAGGTAAAGCGGG	448
Ure-3’	CTTGAATATCCATCTCATCTGC	
rstAB system		
Kinasa int - F	TCAGCTTCATAATCTTCTAG	236
Kinasa int - R	AATAAGATTGTGAGTCTACG	
Tat-pathway protein		
vda-002799-intF	TCCATTTCGTTCTTGTTCGA	1500
vda-002799-intR	GATTTTTCAACGGACGATAT	
Type III secretion system		
vda-000187-intF	GCCGATCTCTTCACCATTAT	140
vda-000187-intR	TAGAACCGCAAAACTGCAAC	
Type III secretion system		
vda-000193-intF	CCAACAATCGCGGCAAGAAT	240
vda-000193-intR	GAGGCTCTTGAATTGTTCTC	
Type III secretion system		
vda-000224-intF	AGTTATAACCGACTTTACTG	511
vda-000224-intR	ACATGCTGGGCGATCAAGC	

Vibrioferrin synthesis PvsB		
DK2_PvsB-1000_F	AGCCTGTGCTCACGAAAGTT	980
DK2_PvsB-1000_R	CGTCATTTTGCCTTCTTTAT	
Vibrioferrin synthesis PvsD		
DK2_PvsD_1200_F	AAATGGTGTTTTGTTGTTGC	1210
DK2_PvsD_1200_R	AATTCTGGAAGCAATTTGGT	
Outer membrane usher		
DK3_outer membrane_800_F	ATTGTGGGATCCTGGTGTTA	800
DK3_outer membrane_800_R	TTTCCATCCATGTTGAGTTT	
Fimbrial protein		
DK3_fimbria_600_F	CGCCTATGTCTATTTTTGGG	600
DK3_fimbria_600_R	GCTACTGCTAACTTTTCCAC	
CRISPR-Cas3(System 1)		
DK29-crispr-CaS3-400-F	AATGCGCTAAATCATAAATC	400
DK29-crispr-Cas3-400-R	AAATCCTTTATGTTGCGTAA	
CRISPR-Csy1 (System 1)		
DK29-crispr-Csy1-200-F	CAGAGCGTTATCAAACTAAG	200
DK29-crispr-Csy1-200-R	ATACTGATTTGATTGGCTCG	
CRISPR-Cas3 (System 2)		
cas3_sist2_F	AAGCTTTGCTACTGACAAGG	1000
cas3_sist2_R	TTAGATCCTTTAAATTCTGT	
CRISPR-Cas2 (System 2)		
cas2_sist2_F	ATCAGCAGGTAAAAATTTCA	150
cas2_sist2_R	TCAGGGAAATGATTTGGGAA	
CRISPR-csy4/cas6 (System 3)		
csy4_sist3_F	TGCGCTGATGAACCGCTAGA	290
csy4_sist3_R	TTGCCTTTGTTGCTGAAGAT	
DK3-hyAch (upstream sequences)		
DK3_contorno_CDS6_F	AACGCTATCATGGGAACTGA	2000
DK3_contorno_CDS7_R	AAATATGTCTAACGGCCTGA	
DK20-hyAch (upstream sequences)		
DK20_contorno_CDS5_R	TCCAAAAGGAGTATTTATCA	2000
DK20_contorno_CDS6_F	GCACCAAGGAGCGGAGCAAA	
DK29-hyAch (upstream sequences)		
DK29_contono_CDS7_R	CAATGTCTGTCCCTGTTAAG	900
HlyAchr_contorno_F	ATAAACAAAAATTAAATTCA	
