# Supplementary material for: Molecular Epidemiology of Photobacterium damselae subsp. damselae Outbreaks in Marine Rainbow Trout Farms Reveals Extensive Horizontal Gene Transfer and High Genetic Diversity
Source: Front Microbiol. 2018 Sep 19;9:2155. doi: 10.3389/fmicb.2018.02155 (PMC6156455; doi:10.3389/fmicb.2018.02155)
Supplement: Supplementary file 2 [file Table_2.PDF]

Supplementary Table S2. Homologies of *P. damselae* subsp. *damselae* proteins encoded by the variable regions downstream *trpR* gene. The six proteins predicted in isolate DK3 and two proteins in isolate DK20 are shown. These proteins are encoded by the variable genes depicted in Figure 6 in the manuscript.

			Homologue Matches			
**Feature ID**	**Length (bp)**	**Function of the predicted protein**	**Accesion no.**	**Species**	**Identities**	**E-value**
DK3_CDS_1	858	Hypothetical protein	WP_104023704.1	*Vibrio hyugaensis*	28%	1e-22
DK3_CDS_2	537	Fimbrial protein precursor	P_025010734.1	*Shewanella* sp.	69%	1e-58
DK3_CDS_3	708	Molecular chaperone	WP_011918440.1	*Shewanella putrefaciens*	40%	8e-54
DK3_CDS_4	2475	Fimbrial biogénesis outer membrane usher protein	WP_011918441.1	*Shewanella putrefaciens*	43%	0.0
DK3_CDS_5	1269	Fimbrial protein	WP_045283300.1	*Shewanella algae*	47%	3e-119
DK3_CDS_6	705	Molecular chaperone	WP_093983961.1	*Shewanella algae*	34%	5e-32
DK20_CDS_1	387	Hypothetical protein	WP_065172226.1	*Photobacterium damselae*	99%	2e-78
DK20_CDS_2	1662	Twin-arginine translocation pathway signal protein	WP_068946980.1	*Photobacterium damselae*	99%	0.0
