# Supplementary material for: Molecular Epidemiology of Photobacterium damselae subsp. damselae Outbreaks in Marine Rainbow Trout Farms Reveals Extensive Horizontal Gene Transfer and High Genetic Diversity
Source: Front Microbiol. 2018 Sep 19;9:2155. doi: 10.3389/fmicb.2018.02155 (PMC6156455; doi:10.3389/fmicb.2018.02155)
Supplement: Supplementary file 3 [file Table_3.PDF]

Supplementary Table S3. Homologies of *P. damselae* subsp. *damselae* proteins encoded by the variable regions upstream and downstream *hlyA*$_{ch}$ gene encoding the hemolysin Phobalysin C. These proteins are encoded by the variable genes depicted in Figure 7 in the manuscript.

			**Homologue Matches**			
**Feature ID**	**Length (bp)**	**Function of the predicted protein**	**Accesion no.**	**Species**	**Identities**	**E-value**
DK2_CDS_1	396	Hypothetical protein	AHL64193.1	*Photobacterium damselae* subsp. *damselae*	98%	2e-90
DK2_CDS_2	414	Hypothetical protein	AHL64227.1	*Photobacterium damselae* subsp. *damselae*	99%	3e-85
DK2_CDS_3	324	His-xaa-ser system protein HxsD	WP_065207553.1	*Photobacterium phosphoreum*	52%	3e-26
DK2_CDS_4	234	Hypotheticalprotein	WP_045038376.1	*Photobacterium* sp.	55%	3e-20
DK2_CDS_5	1461	His-xaa-ser system radical SAM-protein HxsB	OBU47603.1	*Photobacterium phosphoreum*	79%	0.0
DK2_CDS_6	1167	His-xaa-ser system radical SAM-protein HxsB	WP_065207551.1	*Photobacterium phosphoreum*	69%	0.0
DK2_CDS_7	294	Hypothetical protein	WP_065207550.1	*Photobacterium phosphoreum*	56%	1e-05
DK3_CDS_1	396	Hypothetical protein	AHL64193.1	*Photobacterium damselae* subsp. *damselae*	98%	2e-90
DK3_CDS_2	696	Hypothetical protein	WP_065194740.1	*Photobacterium phosphoreum*	59%	3e-85
DK3_CDS_3	537	N-acetyltransferase	WP_021709170.1	*Vibrio azureus*	42%	2e-42
DK3_CDS_4	294	Hypothetical protein	WP_086050176.1	*Vibrio alginolyticus*	55%	6e-25
DK3_CDS_5	1281	Hypothetical protein	WP_086584909.1	*Vibrio parahaemolyticus*	98%	0.0
DK3_CDS_6	828	Integrase	WP_061013430.1	*Photobacterium leiognathi*	99%	0.0
DK3_CDS_7	957	IS110 family transposase	WP_039453501.1	*Vibrio vulnificus*	91%	0.0
DK20_CDS_1	396	Hypothetical protein	AHL64193.1	*Photobacterium damselae* subsp. *damselae*	98%	2e-90
DK20_CDS_2	696	Hypothetical protein	WP_065194740.1	*Photobacterium phosphoreum*	59%	3e-85
DK20_CDS_3	537	N-acetyltransferase	WP_021709170.1	*Vibrio azureus*	42%	2e-42
DK20_CDS_4	294	Hypothetical protein	WP_086050176.1	*Vibrio alginolyticus*	55%	6e-25
DK20_CDS_5	150	Hypothetical protein	WP_081312365.1	*Photobacterium damselae*	81%	0.12
DK20_CDS_6	192	Hypothetical protein	WP_010434923.1	*Vibrio cyclitrophicus*	83%	4e-29
DK20_CDS_7	591	Hypothetical protein	WP_064626907.1	*Vibrio anguillarum*	97%	3e-142
DK29_CDS_1	273	Hypothetical protein	AHL64201.1	*Photobacterium damselae*	93%	2e-52
DK29_CDS_2	312	N-acetyltransferase	WP_045460012.1	*Vibrio hyugaensis*	93%	8e-64
DK29_CDS_3	537	ATP-binding protein	WP_062460271.1	*Vibrio maditerranei*	90%	2e115
DK29_CDS_4	1545	ATP-bindingprotein	WP_071170823.1	*Vibrio cholerae*	88%	0.0
DK29_CDS_5	657	HNH endonuclease	WP_042602772.1	*Vibrio harveyi*	89%	5e-146
DK29_CDS_6	813	Hypothetical protein	WP_010604788.1	*Pseudoalteromonas flavipulchra*	77%	1e-142
DK29_CDS_7	204	Hypothetical protein	WP_010604789.1	*Pseudoalteromonas flavipulchra*	75%	4e-27
DK29_CDS_8	820	HNH endonuclease	WP_068968000.1	*Photobacterium damselae* subsp. *damselae*	99%	0.0
DK29_CDS_9	132	Integrase	WP_045612307.1	*Vibrio vulnificus*	95%	2e-18
DK29_CDS_10	255	Hypothetical protein	WP_081313071.1	*Photobacterium damselae*	94%	3e-48
