# Supplementary material for: Molecular Epidemiology of Photobacterium damselae subsp. damselae Outbreaks in Marine Rainbow Trout Farms Reveals Extensive Horizontal Gene Transfer and High Genetic Diversity
Source: Front Microbiol. 2018 Sep 19;9:2155. doi: 10.3389/fmicb.2018.02155 (PMC6156455; doi:10.3389/fmicb.2018.02155)
Supplement: Supplementary file 4 [file Image_1.PDF]

Phenotype	Genotype	Strains
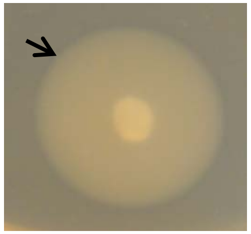	*dly* (phospholipase-D damselysin)<br>*plpV* (Phospholipase-A2)	Strains with pPHDD1 plasmid
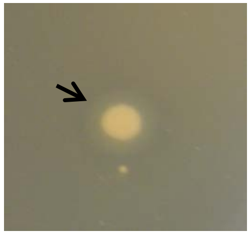	*plpV* (Phospholipase-A2)	Strains without pPHDD1 plasmid

1 cm

Supplementary Figure S1. Phospholipase phenotypes of *P. damselae* subsp. *damselae* strains depending on the presence of one or two phospholipase genes in their genomes. Top pannel: strains harbouring pPHDD1 plasmid, and, therefore, harbouring damselysin gene (*dly*), produce large degradative haloes on TSA-1 plates supplemented with 3% egg yolk extract. Such halo is due to the combined activity of the phospholipase-D damselysin, and the chromosome-encoded phospholipase-A2 encoded by gene *plpV*. Botton pannel: strains without pPHDD1 plasmid do not produce damselysin, and therefore the detected phospholipase activity is due exclusively to the chromosome-encoded PlpV, which is ubiquitous in all the *P. damselae* subsp. *damselae* strains.
