# Supplementary material for: Molecular Epidemiology of Photobacterium damselae subsp. damselae Outbreaks in Marine Rainbow Trout Farms Reveals Extensive Horizontal Gene Transfer and High Genetic Diversity
Source: Front Microbiol. 2018 Sep 19;9:2155. doi: 10.3389/fmicb.2018.02155 (PMC6156455; doi:10.3389/fmicb.2018.02155)
Supplement: Supplementary file 5 [file Image_2.PDF]

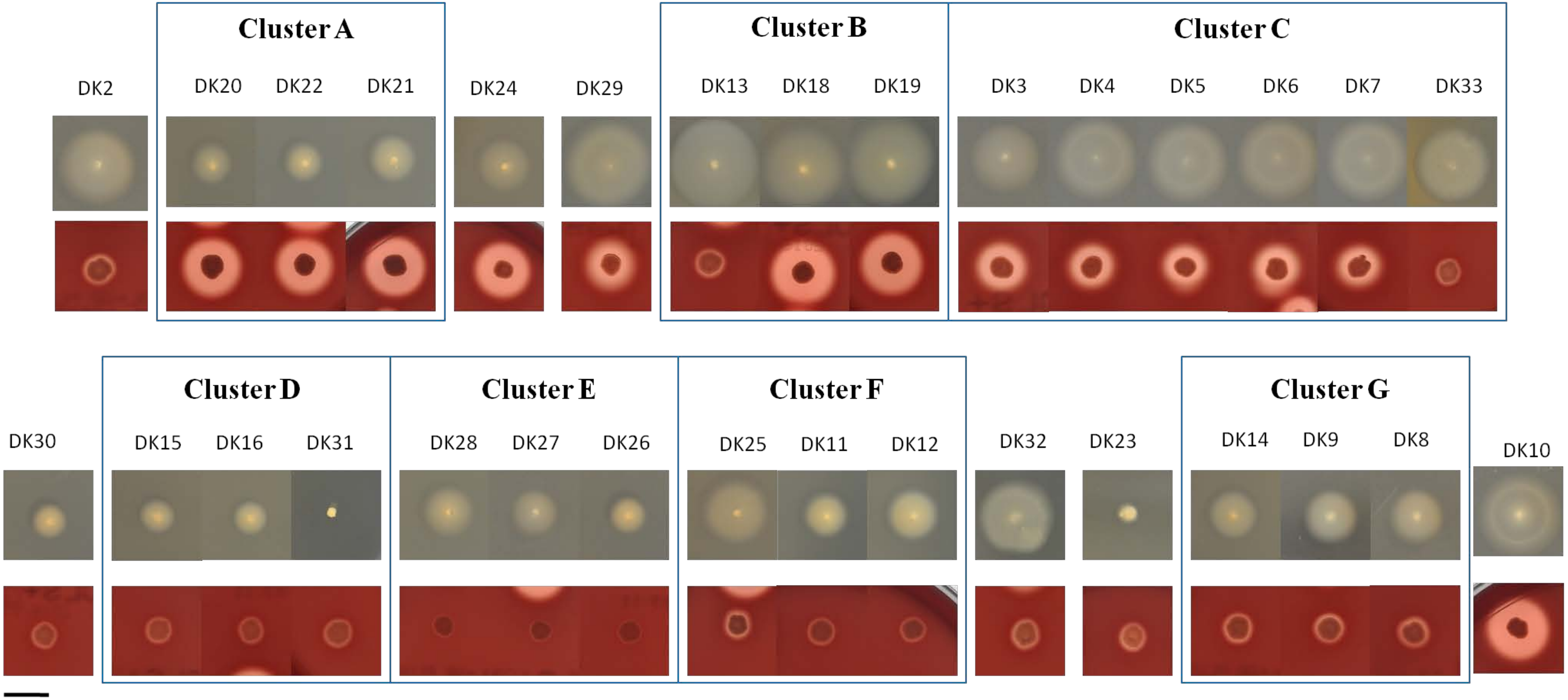

Supplementary Figure S2. Motility (top pictures in each pannel) and hemolysis (botton pictures in each pannel) phenotypes of the 31 *P. damselae* subsp. *damselae* isolates from rainbow trout farms in Denmark analyzed in this study. The horizontal bar at bottom left represents 1 cm scale. The order of the isolates is the same as depicted in the *toxR*-based phylogenetic tree of Figure 1.
