# Supplementary material for: Molecular Epidemiology of Photobacterium damselae subsp. damselae Outbreaks in Marine Rainbow Trout Farms Reveals Extensive Horizontal Gene Transfer and High Genetic Diversity
Source: Front Microbiol. 2018 Sep 19;9:2155. doi: 10.3389/fmicb.2018.02155 (PMC6156455; doi:10.3389/fmicb.2018.02155)
Supplement: Supplementary file 6 [file Image_3.PDF]

A

Strain	Class A beta lactamase	Class C beta lactamase
**DK2**	PSB80478.1	PSB80374.1
**DK3**	PSB78632.1	PSB84194.1
**DK20**	PSB84253.1	PSB89229.1
**DK29**	PSB91507.1	PSB86888.1

B

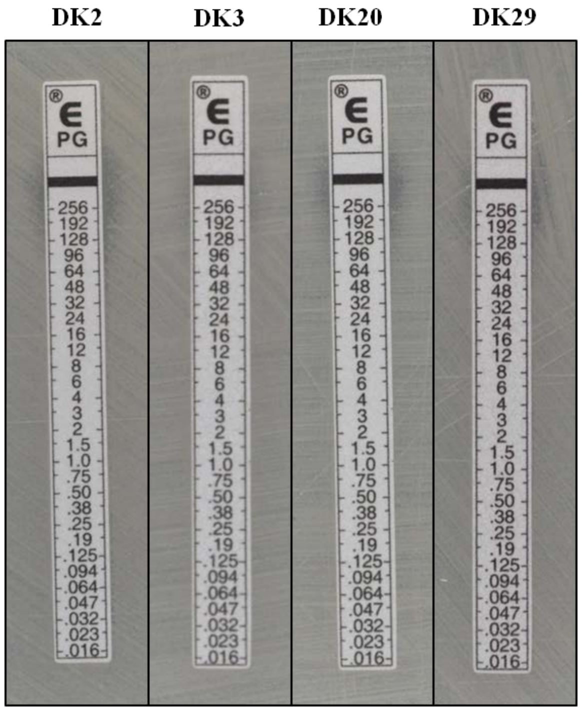

Supplementary Figure S3. **(A)** GenBank Accession numbers of the putative beta lactamase genes encoded within the genomes of *P. damselae* subsp. *damselae* strains DK2, DK3, DK20 and DK29. **(B)** *E*-test for benzylpenicillin sensitivity reveals that the four *P. damselae* subsp. *damselae* strains sequenced in this study are resistant to this antimicrobial. The four strains were found to carry two genes encoding putative β-lactamases within their genomes.
